# Supplementary material for: Nuclear Localization of Human SOD1 in Motor Neurons in Mouse Model and Patient Amyotrophic Lateral Sclerosis: Possible Links to Cholinergic Phenotype, NADPH Oxidase, Oxidative Stress, and DNA Damage
Source: Int J Mol Sci. 2024 Aug 22;25(16):9106. doi: 10.3390/ijms25169106 (PMC11354607; doi:10.3390/ijms25169106)
Supplement: Supplementary file 1 [file ijms-25-09106-s001.zip › ijms-3104716-supplementary.pdf]

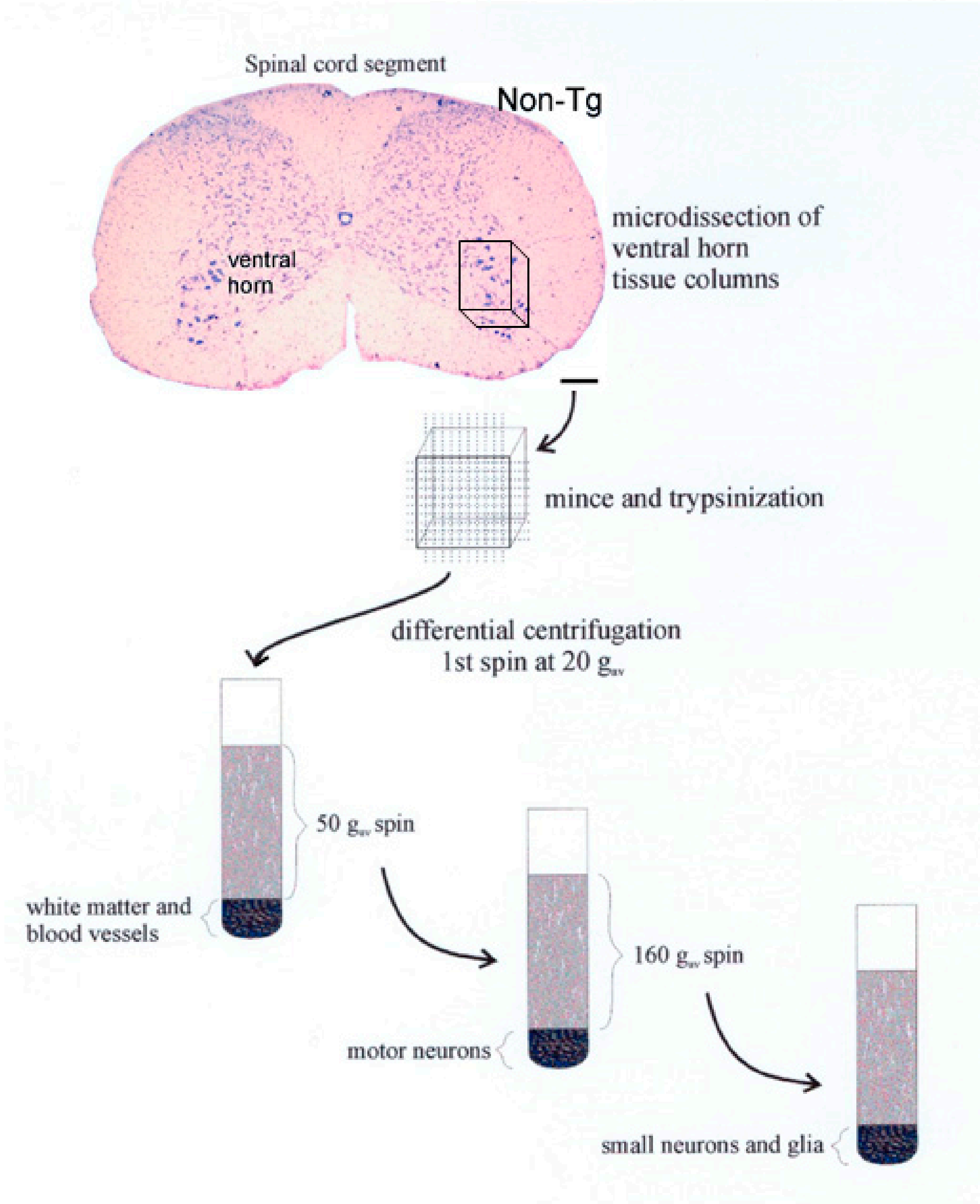


Figure S1. The figure is a schematic diagram of the adult mouse lumbar spinal cord MN isolation procedure.
